# Perceptions and experiences of nondiabetic Cameroonian immigrants in Minnesota on access to affordable, culturally adapted healthcare services

**DOI:** 10.1186/s12913-025-12575-2

**Published:** 2025-05-02

**Authors:** Brendabell Ebanga Njee, Ngambouk Vitalis Pemunta, Vidarah Nimar, Mathias Alubafi Fubah, Asahngwa Constantine Tanywe, Patience Bulage, Tom Obara Bosire, Nguyen Ngoc Bich Tram, Cybel Nji Angwe

**Affiliations:** 1 African Public Health Organization, St. Michael, MN 55376 USA; 2 Department of Sociology, Covenant University, Ota, Nigeria; 3 Cameroon Center for Evidence-Based Health Care, Yaoundé, Cameroon; 4 School of Public Health and Community Medicine, University of Gothenburg, Gothenburg, Västra Götaland County Sweden; 5 Human Sciences Research Council, Developmental, Capable, and Ethical State (DCES) Division, Pretoria, GP South Africa; 6 Centre for Gender and Africa Studies, University of the Free State, Bloemfontein, South Africa; 7 Department of Anthropology, University of Yaoundé 1, Yaoundé, Cameroon; 8 World Bank Group, HAES1, Washington, DC USA; 9 School of Political and Social Sciences, University of Glasgow, Glasgow, Scotland; 10 Budapest Business School, Budapest, Hungary

**Keywords:** Cameroonian immigrants, Behaviors, Beliefs, Knowledge of type 2 diabetes, Cultural competence, Health disparities, Culturally adapted healthcare, Self-management, Immigrant health, Phenomenological study

## Abstract

**Background:**

Cameroonian immigrants, like many ethnic minority groups, face disproportionately high risks of type 2 diabetes due to barriers in accessing culturally responsive and affordable healthcare. This study explores their healthcare experiences, perceptions, and knowledge of diabetes prevention and self-management to identify gaps in healthcare delivery and opportunities for culturally adapted interventions.

**Method:**

Using a phenomenological approach, 13 nondiabetic Cameroonian immigrants were purposively selected for in-depth interviews. Data were transcribed, manually coded, and analyzed using NVivo 14 software to identify recurring themes related to healthcare interactions and diabetes prevention efforts.

**Findings:**

Participants frequently reported leaving medical appointments without fully understanding their healthcare providers’ advice, leading to confusion and poor health outcomes. Many expressed frustration over the lack of integration between psychosocial and physical health needs in their care. Additionally, they emphasized the need for culturally tailored health education, particularly regarding portion control and the nutritional value of traditional foods, to support healthier dietary habits.

**Conclusion:**

This study underscores the urgent need for culturally sensitive healthcare approaches that address the holistic needs of immigrant communities. Strengthening provider-patient communication, fostering trust, and incorporating culturally relevant dietary guidance can enhance diabetes prevention and self-management efforts among Cameroonian immigrants.

**Policy implications:**

Healthcare systems should implement culturally tailored interventions to reduce health disparities and improve provider-patient interactions. Training healthcare providers to adopt a more holistic approach—integrating both psychosocial and physical health considerations—can lead to better health outcomes. Additionally, promoting culturally relevant education on diabetes prevention and management can empower immigrant populations to make informed health decisions and adopt sustainable self-care practices.

**Supplementary Information:**

The online version contains supplementary material available at 10.1186/s12913-025-12575-2.

## Introduction

One of the ambitious targets of the World Health Organization’s (WHO) 2030 Agenda for Sustainable Development is to reduce premature mortality from noncommunicable diseases (NCDs), including diabetes, by one-third while achieving health equity through universal health coverage and access to affordable essential medicines [1]. The WHO Global Burden of Disease Study projects that global diabetes prevalence will rise from 2.8% in 2000 to 4.4% in 2030, with the number of affected individuals increasing from 171 million to 366 million during this period [2]. In 2015, diabetes reportedly caused 50 million deaths worldwide, with a predicted global prevalence of 642 million by 2040 [3].

In the United States, the 2021 National Diabetes Statistics Report indicates that over 38 million Americans, or 11.6% of the population, had diabetes, marking a significant rise from 29 million in 2014. Among them, 8.7 million adults (22.8%) were unaware of their condition [4]. Ethnic minorities, both in the U.S. and globally, face a disproportionately high burden of type 2 diabetes due to socioeconomic disparities, lifestyle factors, genetic predisposition, and inadequate access to culturally competent healthcare. In the U.S., Non-Hispanic Black adults have the highest prevalence at 12.1%, followed by Hispanic adults at 11.8% and Non-Hispanic Asian adults at 9.5%, all notably exceeding the 7.4% recorded for non-Hispanic White adults. These disparities are driven by structural and systemic factors, exacerbating health inequalities [4–6].

The impact of diabetes extends beyond health consequences to substantial economic costs. In the U.S., diabetes-related expenditures surged from $245 billion in 2012 to $327 billion in 2017—a 26% increase. By 2022, these costs surpassed $412.9 billion, underscoring the escalating financial strain on the healthcare system [7, 8]. Ethnic minorities bear a particularly heavy burden, experiencing profound impacts on health, quality of life, healthcare costs, and life expectancy. These populations face higher rates of diabetes-related complications, including kidney failure, retinopathy, stroke, neuropathy, lower limb amputations, and premature death [9, 10]. Additionally, the broader economic impact of health disparities, including those associated with diabetes, underscores the urgent need for targeted interventions to advance health equity [9, 10].

Culturally adapted, cost-effective interventions targeting high-risk migrant groups are critical to mitigating these challenges. Early education on risk factors, promoting physical activity, and dietary interventions are essential to improving clinical outcomes and addressing healthcare disparities [5, 6, 9]. To achieve meaningful results, interventions must address cultural, social, and behavioral barriers specific to diverse populations, such as the experiences of nondiabetic Cameroonian immigrants in accessing healthcare services for type 2 diabetes prevention and management.

## Background

The U.S. population of 328,239,523 represents a growing multiethnic mosaic, posing unique healthcare challenges and necessitating cultural competence among healthcare practitioners [9, 11]. The African immigrant population in the U.S. has grown significantly [11]. Between 2012 and 2022, the number of Black immigrants increased by 23.5%, rising from 3.5 million to 4.3 million [10, 12, 13]. This surge is largely attributed to immigrants from sub-Saharan Africa, whose numbers have increased 16-fold since 1980 [14]. Among these are Cameroonian immigrants residing in Minnesota [15], who, like immigrants from Somalia, Kenya, Ethiopia, Liberia, and Sudan, face health challenges such as obesity and diabetes due to lifestyle changes after living in the U.S. for over five years [15]. The rising prevalence of type 2 diabetes among West African immigrants is attributed to urbanization, dietary changes, and reduced physical activity [16].

Research highlights limited awareness among African immigrants about type 2 diabetes risk factors, with Cameroonians diagnosed at younger ages in developed countries than in their homeland [16]. Studies emphasize the negative health impacts of adopting Western diets, including fast foods [17], while highlighting the benefits of traditional African diets, such as indigenous greens and vegetables, in managing blood sugar [18]. Cultural factors, such as associating obesity with wealth and beauty, further compound diabetes risk [18].

A survey of Cameroonian immigrant diets revealed significant complexity, with 197 recipes identified from 34 commonly consumed foods. The U.S. Department of Agriculture National Nutrient Database analysis showed these diets lacked dairy and included minimal fruit, posing challenges for nutritional assessment [19]. These findings underscore the need for culturally tailored dietary interventions to address diabetes risk in Cameroonian immigrant communities.

Cameroonian immigrants face challenges in understanding and accessing the U.S. healthcare system due to low health literacy rates and language barriers, leaving them vulnerable to diseases like type 2 diabetes. These challenges are compounded by the lack of healthcare policies promoting access for this population [20]. Limited English proficiency further complicates access to preventive care, as many individuals struggle with healthcare materials printed in English, even if educated in their native language [12, 18, 19]. While interpreter services provide some support, Basu et al. [20], advocate for qualified, medically trained interpreters to improve patients’ understanding of their conditions and treatments. However, the presence of multilingual healthcare professionals could further enhance the effectiveness of these interventions [14, 21].

Njeru et al. [22] explored the use of digital storytelling interventions to support African immigrants and refugees with limited English proficiency in managing diabetes. A collaborative approach involving researchers and community members fostered a better understanding of cultural influences on health behaviors. Cultural beliefs significantly shape perceptions and treatment of diabetes [23]. Participants in Njeru and associat’s study [22] often relied on family members for help understanding diabetes and reported cultural barriers such as difficulty giving up traditional foods and managing cravings [23].

This phenomenological study examined (a) access to affordable, quality healthcare services among nondiabetic Cameroonian immigrants in Minnesota and (b) their behaviors, beliefs, and self-management knowledge of type 2 diabetes. Insights into this group’s diabetes-related practices and healthcare access challenges can help providers offer culturally adapted care and improve self-management support.

## Method

 In this study, we acknowledge our positionality as researchers and the ways in which our backgrounds, perspectives, and interactions shaped the research process—from data elicitation to analysis. Our role as cultural insiders or outsiders, depending on individual team members’ backgrounds, influenced participant recruitment, interview dynamics, and the interpretation of findings. By adopting a reflexive approach, we remained cognizant of our influence at each stage of the research [24].

 A purposive sample of 13 non-diabetic Cameroonian immigrants, aged 25–50 and representing both English and French-speaking backgrounds, from the Minneapolis-St. Paul area, voluntarily participated in this study. Recruitment was conducted through flyers distributed at Westminster Presbyterian Church and within the broader Minnesota community. Additionally, we employed snowball sampling, leveraging personal networks and community connections to identify individuals who met specific criteria, including Cameroonian ethnicity, migration to the U.S. after the age of 18, and at least one year of residency in Minnesota. These criteria ensured that participants had established cultural habits, such as dietary practices and health behaviors, before migrating, which could continue to influence their approaches to health management in the U.S. [24–26].

 Semi-structured, open-ended interviews were conducted in participants’ homes, fostering a comfortable setting for discussion. Our positionality influenced the data collection process as we navigated power dynamics, language nuances, and cultural expectations. Some researchers shared cultural and linguistic backgrounds with participants, which facilitated rapport-building and deeper engagement. Others, as outsiders, approached the research with heightened sensitivity to potential biases and the need for trust-building. Probing questions were strategically used to clarify responses, minimize social desirability bias, and ensure consistency across interviews [27–29].

 Throughout data collection, we remained reflexive about our presence and its impact on participants’ narratives. We recognized that our identities could shape how participants framed their experiences, particularly regarding healthcare access, cultural beliefs, and lifestyle adaptations related to type 2 diabetes. As such, we adopted a neutral yet empathetic stance, ensuring that participants felt heard without being led toward specific responses [30, 31].

 To preserve the integrity of participants’ voices, all interviews were audio-recorded with consent and transcribed verbatim. Pseudonyms were assigned to protect confidentiality. Ethical protocols followed National Institutes of Health (NIH) guidelines and received approval from Walden University’s Institutional Review Board (IRB approval number: 01–04–19–049877) [28]. No participants withdrew from the study, and none required counseling services.

 During analysis, we continued to engage in reflexive practice. The transcription process was meticulous, involving multiple rounds of review, cross-referencing field notes, and maintaining fidelity to participants’ original expressions [30, 31]. We employed reflexive thematic analysis (RTA) using NVivo 14—Pro Enterprise (Academic) for Windows, following Braun and Clarke’s six-step framework. As researchers with varying degrees of cultural familiarity, we engaged in continuous dialogue to challenge assumptions and ensure that our interpretations remained grounded in participants’ perspectives rather than our own preconceived notions [29, 32, 33].

 The analysis began with familiarization, where we repeatedly read the transcripts to immerse ourselves in the data and identify emerging patterns. Initial coding involved the application of latent codes, capturing underlying meanings in participants’ narratives [30]. Our positionality played a crucial role here, as we remained vigilant about potential biases that could influence coding decisions. By engaging in team-based coding, we promoted intersubjective validation, refining themes through collaborative discussion** [**28**].**

 As themes were developed, we documented our analytical decisions through an audit trail, ensuring transparency and trustworthiness. Themes were categorized to encapsulate both shared and divergent views on type 2 diabetes among Cameroonian immigrants, [31]. These included Awareness and Knowledge, Beliefs and Perceptions of Type 2 Diabetes, Culturally Appropriate Healthcare, Lifestyle Changes and Adaptation, and Life Changes and Coping Strategies. Reflexivity journaling throughout this process helped us remain attuned to the ways our perspectives shaped our engagement with the data [32].

 By embedding positionality throughout the research process, we acknowledge the complex interplay between researcher and participant, ensuring that our findings are both rigorous and ethically grounded. This reflexive approach not only enhances the credibility of our study but also contributes to more nuanced, culturally responsive understandings of health behaviors among Cameroonian immigrants [33].

## Theoretical framework

This study employed Urie Bronfenbrenner’s Social Ecological Model (SEM) [34] as a guiding framework to explore how multiple levels of influence—individual, interpersonal, organizational, community, and policy—shape health behaviors related to type 2 diabetes prevention. Rather than viewing health as an individual responsibility, SEM emphasizes that behaviors are shaped by social interactions, environmental conditions, and access to coordinated care strategies [34–36]. By considering these interconnected layers, the study examined how family, peers, religious networks, healthcare providers, and policy structures impact diabetes self-management [14, 36, 37].

**Fig. 1:**
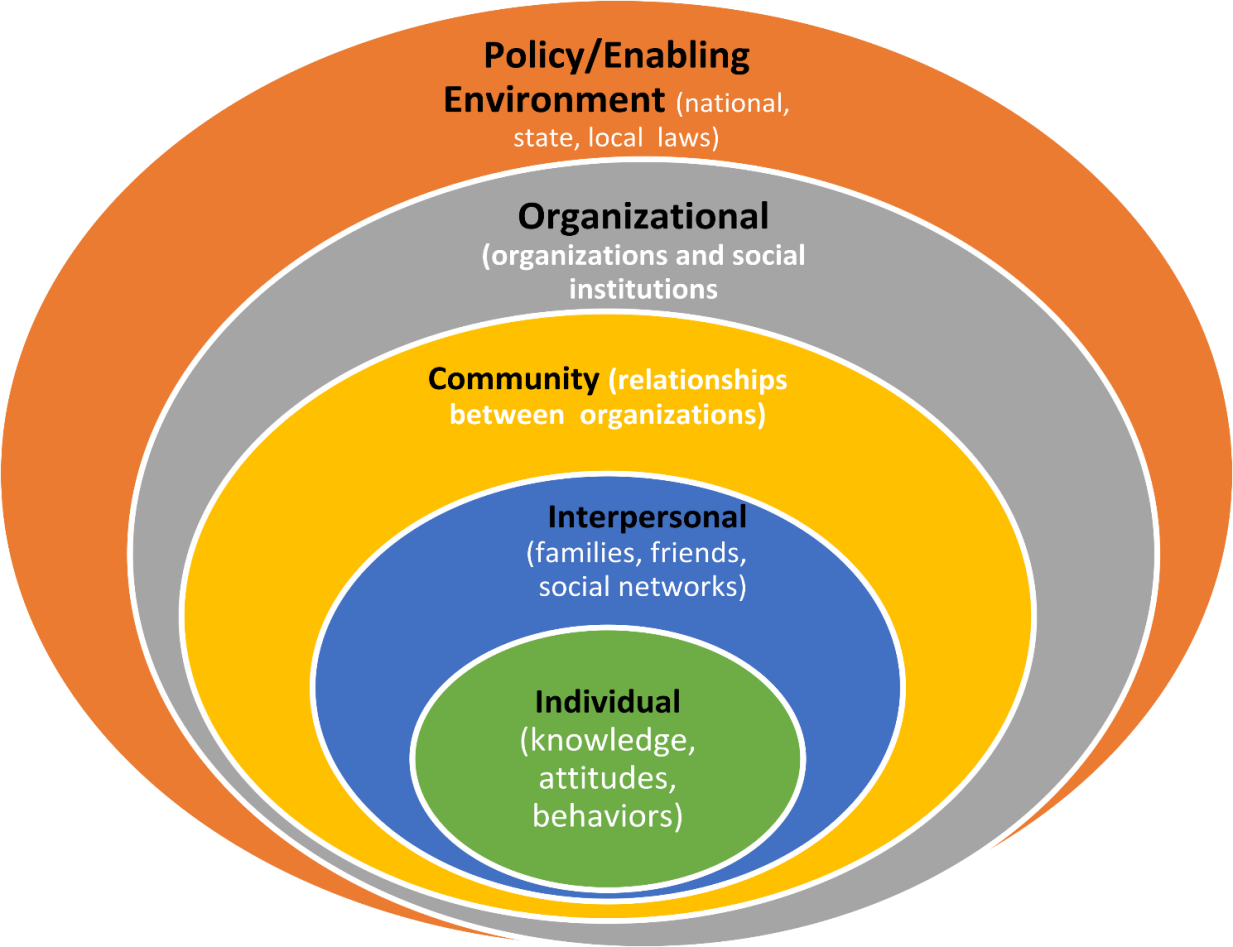
The Social Ecological Model (SEM). (Source: McElfish et al. [36], p. 10)

The framework played a crucial role in shaping the study’s methodology, data analysis, and interpretation of findings. In terms of methodology, SEM guided the research design by ensuring that interviews captured not only individual behaviors but also the broader social and systemic influences affecting participants’ health choices. This approach allowed for a more holistic exploration of diabetes prevention and self-management among nondiabetic Cameroonian immigrants in Minnesota. For data analysis, the findings were categorized according to SEM’s five levels (Fig. 1), providing a structured yet dynamic understanding of the barriers and facilitators shaping diabetes prevention efforts. This layered analysis revealed that diabetes self-management extends beyond individual effort, requiring multilevel collaboration, a notion supported by prior studies showing that interventions addressing multiple levels of influence are more effective than those targeting only one [14, 34–36, 38].

The theoretical framework also shaped the interpretation of findings by highlighting how systemic barriers—such as healthcare access, cultural norms, and policy constraints—hinder effective disease management. Similar to research on Marshallese populations in Springdale, Arkansas, which identified organizational, community, and policy-related obstacles to diabetes self-management [36, 38], this study found that environmental and social factors significantly impact health outcomes. The results further underscored the importance of integrating individual efforts with supportive social networks and structural changes. As Albright [39] emphasizes, tackling the increasing prevalence of type 2 diabetes demands a multifaceted approach, incorporating interventions at every level of human interaction. These range from individual lifestyle modifications and family influences to broader community engagement and policy-driven initiatives. Likewise, integrating diverse behavioral perspectives enhances the comprehension of human-environment dynamics, ultimately fostering more effective policies and resource management strategies [40].

Table 1 outlines the five levels of SEM, illustrating how each level contributes to health behavior and diabetes management.

**Table 1:** A description of social ecological model (SEM) levels [36]

SEM Level	Description
Individual	• Individual level of health-related knowledge, attitudes, health beliefs, perceptions of risk and benefits, values, and preferences [37]. Individual characteristics that influence behaviour change, including knowledge, attitudes, behaviour, self-efficacy, socio-economic status, financial resources, values, goals, expectations, literacy, stigma, and others.
Interpersonal	• The interpersonal level contains patient-centered communication, skills, and social support from formal (and informal) social networks— family and friends, peers, co-workers, religious networks, customs or traditions. The organizational level consists of infrastructure, implementation, and system integration to motivate patients in the self-management of disease.
Community	• At the community level, McCormack et al. encouraged the use of community-based programs, integrated public services, and health care systems [37].
Macro-level Organizational	The macro level features a need to promote public policies, regulations, and incentives. The necessary accountability is grounded in evidence-based strategies [37]. Patient’s health outcome should not rely solely on the individual, but on the engagement of different stakeholders from the five levels of SEM. • Organizations or social institutions with rules and regulations for operations that affect how, or how well, for example, type 2 diabetes services are provided to an individual or groups of individuals.
Policy/Enabling Environment	• Local, state, federal, national and global laws and policies, including policies regarding the allocation of resources for type 2 diabetes and access to healthcare services, restrictive policies (e.g., out of pocket payment for health services), or lack of policies that require early diagnosis for type 2 diabetes [34, 35].

By applying SEM, this study illuminated the interconnected nature of health behaviors and reinforced the need for community-based, multilevel interventions in diabetes prevention. The findings suggest that effective self-management strategies should not only focus on individual behavioral change but also leverage community resources, supportive networks, and policy frameworks to create sustainable health outcomes.

## Findings

This section analyzes the key themes that emerged from exploring Cameroonian immigrants’ knowledge of type 2 diabetes and their access to affordable, quality healthcare services. The first theme, awareness and knowledge, examines their understanding of the disease, including its causes and prevention strategies. Beliefs and perceptions highlight the cultural and individual beliefs surrounding type 2 diabetes and how these shape perceptions of its impact. Experiences and behaviors focus on personal encounters with the condition and the health-related actions taken in response. The theme of lifestyle changes and adaptation explores the adjustments individuals make in their daily habits and how they adapt to new environments or health practices. Motivation and access to care delve into the factors driving health-seeking behavior and the barriers faced in obtaining affordable, quality healthcare. Lastly, perceptions of culturally appropriate healthcare address the extent to which healthcare services are seen as culturally relevant and responsive to their needs.

### Participant demographics

The analyzed demographic data consists of 13 participants, including 11 females and 2 males, with varying experiences of diabetes-related factors. As shown in Table 2, the majority of participants (12 out of 13) have health insurance, with only one participant, Megan, lacking coverage. Approximately half of the participants (6 out of 13) reported a family history of diabetes, with this history observed in 5 females and 1 male, suggesting shared familial or genetic risks. Additionally, 6 paticipants indicated that their friends have a history of diabetes, highlighting the potential role of social networks in shaping awareness or behaviors influencing health outcomes.

**Table 2:** Patient demographics

Pseudonyms	Gender	Health insurance	Family history of diabetes	Friends history of diabetes
Mary	Female	Yes	No	Yes
Magdalene	Female	Yes	Yes	Yes
Martha	Female	Yes	Yes	Yes
Monica	Female	Yes	No	No
Maggie	Female	Yes	No	No
Mirabel	Female	Yes	Yes	No
Melissa	Female	Yes	Yes	No
Molly	Female	Yes	No	No
Megan	Female	No	No	No
Miranda	Female	Yes	No	no
John	Male	Yes	Yes	Yes
Jacob	Male	Yes	No	Yes
James	Male	Yes	Yes	Yes

Source: Fieldwork

Among those with both family and friends’ histories of diabetes, all have health insurance, emphasizing the importance of access to healthcare in populations with heightened diabetes risks. Interestingly, female participants dominate the group without a family or friends’ history of diabetes, potentially reflecting different risk factors compared to males. This demographic profile underscores the interconnected roles of gender, insurance coverage, and social factors in diabetes risk and management.

### Theme 1: awareness and knowledge

Participants’ experiences with health care services and type 2 diabetes prevention reveal evolving awareness and understanding. This theme is organized into four subthemes: Initial Lack of Awareness, Gaining Awareness Through Experiences, Preventive Health Knowledge, and Gaps in Knowledge and Understanding.

#### Subtheme 1: initial lack of awareness

When participants first moved to the U.S., many lacked knowledge about accessing healthcare services and the importance of health insurance. Unlike in Cameroon, where healthcare is predominantly out-of-pocket, navigating the U.S. system posed significant challenges.

- **Miranda**: “As an immigrant, when I first moved here, I did not see health care services as important. When I had my first child, I saw how expensive it was. Then I realized the importance of having insurance.”
- **Molly**: “Here in the United States, you need health insurance, but where I come from in Cameroon, one does not need health insurance to access health care services because you have to pay out of pocket.”

#### Subtheme 2: gaining awareness through experiences

Over time, participants became more familiar with the healthcare system, including the processes of applying for and using health insurance. This increased awareness often stemmed from personal experiences or interactions with healthcare providers.

- **Mary**: “It means I know how to apply and get approved and how to use it.”
- **Melissa**: “When you have access to health care services, you can have enough knowledge about what is going on with you, and you can seek preventive measures on how to take care of yourself.”

#### Subtheme 3: preventive health knowledge

Ten participants reported receiving advice on preventing or managing type 2 diabetes. Common recommendations included dietary changes, regular exercise, and avoiding sugary drinks.

- **James**: “My doctor talked to me about staying away from drinking pop, do exercise, and I have been trying to follow these bits of advice.”
- **Mirabel**: “The health care advice I once had was in Cameroon—that it is not good to overeat starchy food. It is good to eat protein food, do exercise, drink a lot of water, and avoid sugar.”

Participants valued this advice and made efforts to incorporate it into their daily routines.

#### Subtheme 4: gaps in knowledge and understanding

Despite increased awareness, some participants indicated gaps in their knowledge of the healthcare system or type 2 diabetes, revealing areas where additional support or education might be beneficial.

- **Jacob**: “I don’t know, because I do not have too much information on the different types of type 2 diabetes.”
- **Monica**: “I have not worked with someone with diabetes, and I don’t have diabetes, so I do not have a story on that.”

These gaps highlight the ongoing need for accessible and culturally relevant health education to support immigrants in navigating the complexities of healthcare systems and understanding chronic disease prevention.

### Theme 2: knowledge – beliefs and perceptions of type 2 diabetes

In this section, we explore the knowledge and beliefs participants hold about type 2 diabetes, focusing on cultural and individual beliefs, gaps in understanding, and their perceptions of culturally appropriate healthcare. Four key subthemes emerge: Cultural and Individual Beliefs About Type 2 Diabetes, Gaps in Knowledge and Understanding of Type 2 Diabetes, Beliefs About Culturally Appropriate Healthcare and Type 2 Diabetes, and Perceptions of Culturally Appropriate Healthcare and its Impact.

#### Cultural and individual beliefs about type 2 diabetes

Participants’ beliefs about type 2 diabetes were influenced by both cultural norms and personal experiences. Some viewed the disease as primarily linked to lifestyle factors such as diet and exercise, while others associated it with genetic predisposition. For instance, Mary highlighted that type 2 diabetes is linked to high blood sugar and the need for dietary control, while Maggie emphasized the role of obesity and hypertension as contributing factors. Melissa referred to type 2 diabetes as a silent killer that disproportionately affects African Americans, stressing the importance of diet and exercise in managing the condition. Miranda pointed out that type 2 diabetes is often viewed as a lifestyle disease, influenced by eating habits and physical activity. These beliefs reflect the interplay of individual and cultural factors in shaping knowledge about the disease.

#### Gaps in knowledge and understanding of type 2 diabetes

Despite some participants’ awareness, significant knowledge gaps remained among others. For example, Jacob admitted limited understanding of type 2 diabetes, indicating a lack of information on its causes and symptoms. Monica, on the other hand, revealed a complete absence of knowledge, explaining that she had never personally encountered someone with diabetes. Participants like Megan understood type 2 diabetes as a condition primarily managed through diet, but others like Mirabel felt unsure about specific details such as symptoms or causes. These gaps highlight the uneven distribution of knowledge about type 2 diabetes within the group.

#### Beliefs about culturally appropriate healthcare and type 2 diabetes

Participants expressed varying beliefs regarding the importance of culturally appropriate healthcare services. Some saw culturally sensitive care as essential, emphasizing the need for healthcare providers to understand cultural backgrounds and offer patient-centered care. John noted the importance of healthcare providers knowing their cultural background to help patients freely communicate about their dietary habits and lifestyle choices. Magdelene shared the view that culturally appropriate care involves considering cultural traditions and practices, fostering confidence in receiving treatment. Maggie mentioned that healthcare services need to respect religious beliefs and dietary restrictions. In contrast, others related culturally appropriate care to the use of traditional remedies, which they felt were often overlooked by healthcare providers.

#### Perceptions of culturally appropriate healthcare and its impact

Participants’ perceptions of culturally appropriate healthcare varied. Some believed they received culturally tailored care, while others felt these services were only sometimes or rarely culturally appropriate. Mary shared that her Christian faith was respected by healthcare providers, enhancing her confidence in their care. Jacob acknowledged that providers sometimes inquired about cultural beliefs affecting treatment decisions. Maggie highlighted the growing awareness in healthcare of diverse cultural backgrounds and dietary practices. However, participants like Mirabel and Melissa expressed a more mixed experience, noting that culturally appropriate care was not consistently provided. Mirabel, for example, described her experience as 50/50, while Melissa emphasized that providers who respected cultural practices, such as prayer schedules or dietary preferences, offered more supportive care.

These subthemes underscore the complex relationship between cultural beliefs, gaps in knowledge, and perceptions of culturally appropriate healthcare in shaping participants’ understanding and management of type 2 diabetes.

### Theme 4: lifestyle changes and adaptation

Participants reported a variety of lifestyle changes and adaptations since accessing healthcare in Minnesota, which have contributed to improved health outcomes. These changes primarily involve adjustments to daily habits, such as dietary practices and physical activity, as well as adaptation to new health practices and environments. Below, we highlight key insights and quotes from the interviews that illustrate how participants have navigated these changes.

#### Jacob

“I would say my health has improved because I have access to health care. I can see my health care provider every six months for regular check-ups, and I would think my health is improving because if anything is coming up just for the fact that I see my health care provider every six months, that can be addressed.”

Jacob’s quote highlights how access to healthcare has facilitated regular check-ups, helping him manage potential health issues before they escalate. His engagement with healthcare providers has enabled him to adopt a proactive approach to monitoring and maintaining his health.

#### Magdalene

“I will say yes, it had improved because when I first came here and went to the hospital, I was introduced to so many things that I had never used. Especially, when I first got here, I was approaching 40 years, and I was advised to do certain tests that I wasn’t even aware of, like eye examination, sugar level, and high blood pressure. I think they have improved. I have learned to be proactive with my health. I must see my primary care provider at least once a year. This has helped me to manage some of the illnesses that I had and some that I did not know that I had by seeing her frequently.”

Magdalene’s experience emphasizes the importance of regular health screenings, which uncovered unknown health issues. Her proactive engagement with her primary care provider has been critical in managing both known and previously undiagnosed conditions.

#### Miranda

“I take my yearly check-ups seriously, and I make sure I go for this check-up to make sure something is not developing. Yes, I have been fortunate to have doctors that understand me and are encouraging.”

Miranda’s statement reinforces the value of yearly check-ups and the role of supportive healthcare providers in fostering a sense of confidence and proactive health management.

#### Maggie

“Dietary behavior includes the way you eat. Activity behaviors include exercise, like going to the gym. Both dietary and activity behaviors can affect your risk of type 2 diabetes when you do not control your exercise and diet.”

Maggie highlights how awareness of dietary and activity behaviors has led to significant changes in her daily life, particularly through regular physical activity and attention to what she eats, both of which have been essential in reducing her risk of type 2 diabetes.

The above quotes illustrate how access to healthcare, regular check-ups, and an increased focus on dietary and physical activity behaviors have collectively contributed to positive health adaptations and improved self-management practices among participants.

Furthermore, several participants reported being motivated to make lifestyle and behavioral changes, particularly in reducing carbohydrate and sugar intake. Jacob noted the external influence of his wife in encouraging healthier eating habits:

John; “My wife is the one who encourages me to eat healthily. She has really pushed me to make changes in my diet.”

Others reported increased motivation to exercise and improve their diet by incorporating more fruits and vegetables into their daily routines. For example:

Molly; “I have started eating more fruits and vegetables and exercising more because I know it helps with my health.”

These motivations highlight both internal drive and external influences, such as family support, that shaped participants’ efforts to modify their diet and exercise habits. Their commitment to adopting healthier routines reflects a proactive approach to managing their well-being and addressing health concerns, including type 2 diabetes.

### Theme 5: lifestyle changes, adaptation, and perceptions

Participants highlighted various aspects of lifestyle changes, adaptation to new environments, and their perceptions of culturally appropriate healthcare services. Below, we merge themes related to lifestyle changes and adaptation, incorporating insights into perceptions of culturally appropriate care.

#### Lifestyle changes and adaptation

Many participants described adapting to the American healthcare system and lifestyle practices to access the services they need. Martha’s experience illustrates the tension between cultural traditions and new health practices:

Martha; “It was not appropriate to my culture. It is culturally appropriate to the Americans, so I have decided to adapt to the American ways to get the services that I need, but from my culture, the answer is no.”

John; “They don’t understand my culture” and “Doctors don’t care about our traditions”.

Similar to Martha’s, John’s reflection highlights the complexity of balancing cultural values with the practical need for healthcare access. These quotes highlight the complex relationship between culture and healthcare access, particularly in the context of diabetes care. These respondents acknowledge a tension between their cultural background and the expectations of the American healthcare system, ultimately choosing to adapt in order to receive necessary services. This decision underscores the challenges faced by individuals from diverse cultural backgrounds when navigating healthcare systems that may not fully accommodate their beliefs, values, and practices.

A nuanced discussion of culture in this context recognizes that it is not a static or monolithic concept but rather dynamic and adaptable. Culture shapes health beliefs, dietary practices, perceptions of illness, and attitudes toward medical interventions. For instance, some cultures may prioritize traditional or herbal remedies over biomedical treatments, view certain dietary restrictions as impractical, or perceive medical authority differently than the dominant healthcare model. In diabetes care, cultural considerations may influence food choices, medication adherence, attitudes toward insulin use, and health literacy.

Similarly, some participants initially struggled to understand the importance of health insurance but eventually adapted as they encountered costly healthcare experiences. Miranda shared:

“When I had my first child, I saw how expensive it was, then I realized the importance of having insurance.”

This quote underscores the gradual acceptance of the American healthcare system as immigrants navigate new financial realities.

Molly described the difficulty of adapting to dietary changes due to cultural food preferences:

“It becomes challenging when you come over here in the United States, and you can no longer eat some of these delicacies. Delicacies to me mean the cultural food that I grew up eating, such as ekwang, khatikhati, and ndole.” Molly’s experience reflects the struggles of maintaining cultural food habits in a new environment, especially when faced with unfamiliar dietary options.

Molly: “I reduced sugar and carbohydrates. I try to eat more fruits and vegetables”.

These statements indicate conscious dietary adjustments aimed at better health management. Reducing sugar and carbohydrates suggests an effort to control blood sugar levels, possibly to prevent or manage diabetes. Increasing fruit and vegetable intake reflects a shift toward a more balanced diet, likely to improve overall nutrition and well-being.

### Theme 6: perceptions of culturally appropriate healthcare

Participants expressed differing views on culturally appropriate healthcare and its relevance to their health experiences in Minnesota. James noted a significant shift in his healthcare habits:

“Access to health care services means a lot to me compared to Cameroon, where I came from. I go for yearly check-ups, which I never did back home. I am aware of what I am eating, which back home we go with what we have.” James’ reflections highlight the contrast between his experiences in Cameroon and Minnesota, emphasizing the role of healthcare access in promoting awareness and proactive health management.

John, on the other hand, expressed limited engagement with culturally specific practices in Minnesota:

“I can’t think of anything culturally that has helped me here to stay healthy, because people just want to understand where you come from, the food you ate when you were young, or back home cultural dishes, apart from that, nothing else culturally.” John’s perspective suggests that, despite recognizing cultural connections to food, healthcare access and cultural support may not have played a significant role in his health outcomes.

These findings reflect the diversity of experiences among participants, with some adapting to the American healthcare system while navigating tensions between cultural traditions and new health practices.

## Discussion of findings

The purpose of this qualitative phenomenological study was to explore how nondiabetic Cameroonian immigrants in Minnesota access affordable, quality healthcare services, as well as their behaviors, beliefs, and self-management knowledge of type 2 diabetes. By examining their perceptions and experiences with healthcare access and type 2 diabetes awareness, this study aimed to identify healthcare gaps and inform culturally responsive interventions. The findings contribute to improving healthcare access for Cameroonian immigrants and raising public awareness about type 2 diabetes incidence within this vulnerable group.

 This section discusses the study’s findings through the lens of the Social Ecological Model (SEM), including themes identified in participant responses, connections to existing literature, limitations, implications, conclusions, and recommendations. The SEM framework incorporates intrapersonal, interpersonal, organizational, community, and public policy levels [35–40] to highlight how health outcomes are shaped by individual behaviors, social interactions, and systemic structures.

### Perceptions of access to healthcare for type 2 diabetes self-management

#### (A) Awareness and knowledge (intrapersonal level)

Participants expressed limited awareness of healthcare access and type 2 diabetes risk factors. This lack of awareness aligns with findings from Zhang et al. [23], emphasizing the need for culturally tailored health education. The intrapersonal level of SEM underscores the significance of individual knowledge and health literacy in shaping self-management behaviors [35, 36, 40].

Despite some familiarity with the U.S. healthcare system, many participants lacked a clear understanding of health insurance and preventive care, preferring traditional healing practices learned in Cameroon. This supports Choukem et al. [15], who found that cultural shifts influence diabetes risk among African immigrants. Furthermore, Wafula and Snipes [20] highlight that cultural incongruence between healthcare providers and African immigrant patients contributes to barriers in healthcare access. Many respondents reported negative experiences with healthcare providers, reinforcing the need for culturally competent care.

#### (B) Beliefs and perceptions (interpersonal level)

Beliefs about healthcare and diabetes varied among participants. Some expressed skepticism about Western medicine, referring to pharmaceutical treatments as “pill popping” and questioning healthcare providers’ cultural competence. Others recognized the necessity of medical intervention but preferred integrating herbal remedies and traditional practices. This reflects the interpersonal level of SEM, where family, friends, and cultural beliefs influence health behaviors [34].

McCormack et al. [37] emphasize the role of social support in effective diabetes self-management. However, this study revealed a lack of sufficient interpersonal support among participants, contributing to their challenges in navigating the healthcare system. Many respondents reported feeling misunderstood by providers due to language barriers and accents, leading to avoidance of routine check-ups. This supports Wafula and Snipes [20], who link healthcare delays to providers’ lack of cultural awareness.

#### (C) Experiences and behaviors (organizational level)

The organizational level of SEM examines how healthcare institutions influence patient experiences [34, 35]. Many participants expressed feeling that healthcare providers lacked cultural sensitivity, noting that their cultural background was not understood or acknowledged. Some shared sentiments such as, “They don’t recognize our culture,” and “Doctors disregard our traditions.” Religious beliefs also played a role, with respondents noting that providers failed to acknowledge their faith-based perspectives on healing. These findings align with Franz et al. [41], who emphasize the importance of holistic, culturally responsive healthcare in diabetes self-management.

Participants expressed a strong preference for providers who demonstrate cultural competence and willingness to integrate traditional healing perspectives into care plans. This supports the argument that healthcare systems should incorporate multicultural training for providers to improve patient-provider interactions [14].

#### (D) Lifestyle changes and adaptation (community level)

Many respondents actively sought to modify their diets and exercise habits to prevent diabetes and obesity. Participants expressed making dietary adjustments, stating that they had decreased their intake of sugar and carbohydrates while making an effort to consume more fruits and vegetables. This reflects a willingness to adapt despite challenges in accessing healthy food options, reinforcing Powers et al. [42], who emphasize the role of dietary and physical activity interventions in managing chronic diseases. Cultural sensitivity is vital for effective diabetes care in low-income, diverse populations, as it builds trust, enhances communication, and supports treatment adherence [43]. Improving cultural competence in healthcare can strengthen diabetes management, encourage healthier behaviors, and reduce disparities [44].

However, participants faced significant barriers, including limited access to affordable, nutritious food and culturally relevant health resources, a challenge also highlighted by Sallis et al. [34]. Respondents reported difficulty finding traditional foods in Minnesota, with some resorting to importing ingredients from Cameroon. The community level of SEM highlights how social networks and neighborhood resources shape health behaviors [34, 35]. Participants expressed that support groups and culturally relevant health programs would improve adherence to lifestyle changes. This aligns with McCormack et al. [37], who emphasize that community-based interventions enhance diabetes prevention efforts.

#### (E) Motivation and access to care (public policy level)

Participants reported that structural barriers within the U.S. healthcare system hindered their access to quality care. They highlighted issues such as high medical costs, insurance complexities, and a lack of culturally competent providers. Many noted that while insurance improved affordability, it did not guarantee culturally appropriate care. This finding aligns with Issaka et al. [45], who observed that African immigrants in Melbourne faced systemic barriers to preventive healthcare, contributing to increased diabetes risk.

The public policy level of SEM emphasizes the role of government policies in shaping healthcare access and health equity [34, 35]. Participants expressed frustration with a reactive healthcare model, stating that providers focused on treating illness rather than prevention. They also noted that limited public health outreach efforts targeted African immigrant communities, reinforcing existing disparities. Addressing these gaps requires policy interventions that prioritize culturally responsive care and community engagement.

### Limitations of the study

While this study provides valuable insights into the experiences of nondiabetic Cameroonian immigrants in Minnesota, certain limitations must be acknowledged. First, the findings cannot be generalized to the entire population of nondiabetic Cameroonian immigrants in Minnesota, as the sample consisted of only 13 individuals aged 25 to 50, drawn from a single geographic location. The focus on Minnesota further restricts the study’s applicability to Cameroonian immigrants in other U.S. states or broader diaspora communities.

Second, due to extreme weather conditions that limited participants’ mobility, interviews were conducted in their homes. While this setting may have fostered comfort and openness, it also introduced potential biases, such as responses influenced by the home environment or the presence of family members.

Third, the small sample size, though appropriate for qualitative research, may not capture the full diversity of perspectives within the target population. While qualitative research prioritizes depth and richness of data over sheer numbers [46], a larger sample could enhance the robustness and transferability of findings. Future studies might employ a mixed-methods or quantitative approach to broaden representation and increase generalizability.

Additionally, participant recruitment relied on individuals expressing interest in the study and voluntarily reaching out to the research team. This self-selection process may have introduced selection bias, as those with strong opinions or particular experiences may have been more inclined to participate.

Finally, as with any qualitative study, researcher positionality and potential biases in data interpretation must be considered. Despite efforts to ensure rigor and reflexivity, the researcher’s background, assumptions, and interactions with participants may have shaped the findings in subtle ways. Future research could mitigate these concerns by incorporating multiple researchers, triangulating data sources, or employing member-checking techniques.

Despite these limitations, the study offers a meaningful contribution to understanding the lived experiences of nondiabetic Cameroonian immigrants in Minnesota, providing a foundation for future research in this area.

This study reveals that healthcare access and diabetes prevention among Cameroonian immigrants are shaped by a complex interplay of individual knowledge, cultural beliefs, healthcare experiences, community resources, and systemic policies. The Social Ecological Model (SEM) provided a comprehensive framework to examine these multilevel influences. Key findings include:

Limited awareness and health literacy at the intrapersonal level create significant barriers to diabetes prevention efforts. Cultural and religious beliefs further shape health-seeking behaviors, influencing how individuals approach care and treatment. At the organizational level, many healthcare institutions lack cultural competence, often leading to negative patient experiences and diminished trust in the healthcare system. Within communities, access to culturally relevant health resources and support networks plays a crucial role in promoting and sustaining lifestyle changes. Meanwhile, systemic barriers at the public policy level, including high healthcare costs and policy gaps, continue to impede equitable access to diabetes prevention and management services.

 A central insight from this study is that culturally appropriate healthcare for diabetes management requires more than translation services or superficial acknowledgment of diversity. Culture is not a static barrier but a dynamic factor that shapes health behaviors, dietary choices, and engagement with medical care. The experience of Cameroonian immigrants highlights the need for healthcare systems to move beyond expecting patients to adapt to dominant medical norms and instead integrate cultural sensitivity into service delivery. This includes incorporating familiar dietary recommendations, involving community leaders in health education, and fostering trust through culturally competent providers. By addressing cultural and structural barriers simultaneously, healthcare systems can reduce the burden of adaptation placed on immigrant populations and improve health outcomes through equitable, patient-centered care.

## Recommendations

To improve diabetes prevention and healthcare accessibility, it is essential to increase culturally tailored health education initiatives that raise awareness and empower individuals to make informed health decisions. Healthcare providers should receive enhanced training in cultural competency to foster better patient-provider relationships, ensuring more effective communication and trust-building. Additionally, expanding community-based health interventions that blend traditional practices with modern healthcare can create a more supportive environment for sustained lifestyle changes. Finally, advocating for policy reforms that improve healthcare access for African immigrant populations is critical to addressing systemic barriers and ensuring equitable care.

### Moving forward

Expanding culturally tailored health education, strengthening provider training, enhancing community-based interventions, and advocating for policy reforms can create more inclusive healthcare strategies. A multilevel SEM approach will help healthcare systems develop equitable, patient-centered solutions for diabetes prevention and management among Cameroonian immigrants in Minnesota and beyond.

By addressing these challenges through a multilevel SEM approach, healthcare systems can create more inclusive, effective strategies for diabetes prevention and management among Cameroonian immigrants in Minnesota and beyond.

## Supplementary Information

Supplementary Material 1.

## Data Availability

The datasets generated and/or analyzed during this study are not publicly available due to the presence of participant identifiers that could potentially compromise their confidentiality and well-being. However, the datasets (interview transcripts) are available from the corresponding author upon reasonable request.

## Abbreviations

ADA: American Diabetes Association
CDC: Centers for Disease Control and Prevention
IRB: Institutional Review Board
NCDs: Noncommunicable diseases
SEM: Social Ecological Model
USA: United States of America
US: United States
WHO: World Health Organization

## References

[1] World Health Organization (WHO). Global Report on Diabetes. France: WHO; 2016.

[2] Wild S, Roglic G, Green A, et al. Global prevalence of diabetes: estimates for the year 2000 and projections for 2030. Diabetes Care. 2004;27(5):1047–53. 10.2337/diacare.27.5.1047. PMID: 15111519. 15111519 10.2337/diacare.27.5.1047

[3] Ogurtsova K, da Rocha Fernandes JD, Huang Y, et al. Unsatisfactory saturation’: A critical exploration of the notion of saturated sample sizes in qualitative research. Qualitative Res. 2012;13(2):190–7. 10.1177/1468794112446106.

[4] Centers for Disease Control and Prevention (CDC). National Diabetes Statistics Report: Estimates of Diabetes and Its Burden in the United States, 2021. Centers for, Control D, Prevention. U.S. Department of Health and Human Services.

[5] Caballero AE. The ‘A to Z’ of managing type 2 diabetes in culturally diverse populations. Front Endocrinol. 2018;9:479. 10.3389/fendo.2018.00479. 10.3389/fendo.2018.00479 PMC6127640 30233490

[6] Bennet LH. Diabetes risk bland icke-västerländska invandrare - Riktad och strukturerad prevention behövs [Ethnic and cultural aspects of type 2 diabetes]. Lakartidningen. 2018;115:EWPF Swedish. PMID: 29461580. 29461580

[7] American Diabetes Association. Economic costs of diabetes In the U.S. In 2017. Diabetes Care. 2018;41(5):917–28. 10.2337/dci18-0007. 29567642 10.2337/dci18-0007 PMC5911784

[8] MarketWatch. Surging diabetes rate underscores vast opportunity in weight-loss drugs. 2023. Retrieved from https://www.marketwatch.com/story/surging-diabetes-rate-underscores-vast-opportunity-in-weight-loss-drugs-c7d37bfd.

[9] Haw JS, Shah M, Turbow S, Egeolu M, Umpierrez G. Diabetes complications in Racial and ethnic minority populations in the USA. Curr Diab Rep. 2021;21(1):2. 10.1007/s11892-020-01369-x. PMID: 33420878; PMCID: PMC7935471. 33420878 10.1007/s11892-020-01369-x PMC7935471

[10] LaVeist TA, Pérez-Stable EJ, Richard P, Anderson A, Isaac LA, Santiago R, Okoh C, Breen N, Farhat T, Assenov A, Gaskin DJ. The Economic Burden of Racial, Ethnic, and Educational Health Inequities in the US. JAMA. 2023;329(19):1682–92. 10.1001/jama.2023.5965. Erratum. in: JAMA. 2023;329(21):1886. 10.1001/jama.2023.9136. PMID: 37191700. 37191700 10.1001/jama.2023.5965

[11] Gambino CP, Trevelyan EN, Fitzwater JT. Foreign-born Population from Africa, 2008-2012. US Department of Commerce, Economic and Statistics Administration, US Census Bureau. 2014. Retrieved from https://www.immigrationresearch.org/system/files/africa_population_foreign_born.pdf.

[12] U.S. Census Bureau. QuickFacts: United States. U.S. Department of Commerce. 2019. https://www.census.gov/quickfacts/fact/table/US.

[13] Sewali B, Harcourt N, Everson-Rose SA, et al. Prevalence of cardiovascular risk factors across six African immigrant groups in Minnesota. BMC Public Health. 2015;15(1):411. 10.1186/s12889-015-1740-3. 25895917 10.1186/s12889-015-1740-3 PMC4409770

[14] Zong Jie, Jeanne Batalova. "Sub-Saharan African Immigrants in the United States." Migration Policy Institute. 2020. https://www.migrationpolicy.org/article/sub-saharan-african-immigrants-united-states-2019.

[15] Choukem SP, Fabreguettes C, Akwo E, et al. Influence of migration on characteristics of type 2 diabetes in sub-Saharan Africans. Diabetes Metab. 2014;40(1):56–60. 10.1016/j.diabet.2013.07.004. 24076360 10.1016/j.diabet.2013.07.004

[16] Sharma S, Claude Mbanya J, Cruickshank K, et al. Nutritional composition of commonly consumed composite dishes from the central Province of Cameroon. Int J Food Sci Nutr. 2007;58(6):475–85. 10.1080/09637480701288454. 17710591 10.1080/09637480701288454

[17] Ilunga Tshiswaka D, Ibe-Lamberts KD, Mulunda DM, et al. Perceptions of dietary habits and risk for type 2 diabetes among Congolese immigrants. J Diabetes Res. 2017;2017:4736176. 10.1155/2017/4736176. 29259994 10.1155/2017/4736176 PMC5702411

[18] Wafula EG, Snipes SA. Barriers to health care access faced by black immigrants in the US: theoretical considerations and recommendations. J Immigr Minor Health. 2014;16(4):689–98. 10.1007/s10903-013-9898-1. 24006174 10.1007/s10903-013-9898-1

[19] Juckett G, Unger K. Appropriate use of medical interpreters. Am Fam Physician. 2014;90(7):476–80. 25369625

[20] Basu G, Costa VP, Jain P. Clinicians’ obligations to use qualified medical interpreters when caring for patients with limited english proficiency. AMA J Ethics. 2017;19(3):245–52. 10.1001/journalofethics.2017.19.3.ecas2-1703. 28323605 10.1001/journalofethics.2017.19.3.ecas2-1703

[21] Zeh P, Sandhu H, Cannaby AM, Sturt J. The impact of culturally competent diabetes care interventions for improving diabetes-related outcomes in ethnic minority groups: a systematic review. Diabet Med. 2012;29(10):1237–52. 10.1111/j.1464-5491.2012.03701.x. 22553954 10.1111/j.1464-5491.2012.03701.x

[22] Njeru JW, Patten CA, Hanza MM, et al. Stories for change: development of a diabetes digital storytelling intervention for refugees and immigrants to Minnesota using qualitative methods. BMC Public Health. 2015;15(1):1311. 10.1186/s12889-015-2628-y. 26715465 10.1186/s12889-015-2628-y PMC4696160

[23] Haas L, Maryniuk M, Beck, et al. National standards for diabetes self-management education and support. Diabetes Care. 2013;36(Supplement 1):S100–8. 10.2337/dc13-S100. 23264420 10.2337/dc13-S100 PMC3537270

[24] O’Brien MJ, Shuman SJ, Barrios DM, et al. A qualitative study of acculturation and diabetes risk among urban immigrant Latinas: implications for diabetes prevention efforts. Diabetes Educ. 2014;40(5):616–25. 10.1177/0145721714535992. 24872386 10.1177/0145721714535992 PMC4169339

[25] Etikan I, Alkassim R, Abubakar S. Comparison of snowball sampling and sequential sampling technique. Biom Biostat Int J. 2016;3(1):55–6. 10.15406/bbij.2015.03.00055.

[26] Dickson VV, McCarthy MM, Howe A, et al. Sociocultural influences on heart failure self-care among an ethnic minority black population. J Cardiovasc Nurs. 2013;28(2):111–8. 10.1097/JCN.0b013e31823db328. 22343210 10.1097/JCN.0b013e31823db328

[27] Zhang X, Beckles GL, Bullard KM, Gregg EW, Albright AL, Barker L, Zhang X, Ruiz-Holguín R, Cerqueira MT, Frontini M, Imperatore G. Access to health care and undiagnosed diabetes along the united States-Mexico border. Revista Panam De Salud Pública. 2010;28(3):182–9. 10.1590/s1020-49892010000900008 20963265

[28] Ramani S, Mann K. Introducing medical educators to qualitative study design: twelve tips from inception to completion. Med Teach. 2016;38(5):456–63. 10.3109/0142159X.2015.1035244. 25897710 10.3109/0142159X.2015.1035244

[29] Miles MB, Huberman AM, Saldana J. Qualitative data analysis. Thousand Oaks: SAGE; 2013.

[30] Graue C. Qualitative data analysis. Int J Sales Retail Mark. 2015;4(9):5–14. Retrieved from www.ijsrm.com/ijsrm/current_&past_issues_files/ijsrm4-9.

[31] Sutton J, Austin Z. Qualitative research: Data collection, analysis, and management. Can J Hosp Pharm. 2015;68(3):226. Retrieved from https://www.ncbi.nlm.nih.gov/pmc/articles/PMC4485510/. 26157184 10.4212/cjhp.v68i3.1456 PMC4485510

[32] Flick U. An introduction to qualitative research. Thousand Oaks: SAGE; 2014.

[33] Creswell JW, Creswell JD. Research design: qualitative, quantitative, and mixed methods approach. Thousand Oaks: SAGE; 2017.

[34] Bronfenbrenner U. Toward an experimental ecology of human development. Am Psychol. 1977;32(7):513–31. Retrieved from https://pdfs.semanticscholar.org/.

[35] Sallis JF, Owen N, Fisher E. Ecological models of health behavior. In: Glanz K, Rimer BK, Viswanath K, editors. Health behavior: theory, research, and practice. Hoboken: Wiley; 2015. p. 43–64.

[36] McElfish PA, Moore R, Woodring D, et al. Social ecology and diabetes self-management among Pacific Islanders in Arkansas. J Family Med Disease Prev. 2016;2(1). 10.23937/2469-5793/1510026. 10.23937/2469-5793/1510026 PMC5518699 28736764

[37] McCormack L, Thomas V, Lewis MA, et al. Improving low health literacy and patient engagement: A social-ecological approach. Patient Educ Couns. 2017;100(1):8–13. 10.1016/j.pec.2016.07.007. 27475265 10.1016/j.pec.2016.07.007

[38] Fisher EB, Brownson CA, O’Toole ML, et al. Ecological approaches to self-management: the case of diabetes. Am J Public Health. 2005;95(9):1523–35. Retrieved from https://ajph.aphapublications.org/doi/pdfplus/10.2105/AJPH.2005.066084. 16051929 10.2105/AJPH.2005.066084 PMC1449392

[39] Albright C. Social ecological models and their impact on health outcomes. J Health Behav Social Sci. 2015;12(3):245–58.

[40] Schlüter M, Baeza A, Dressler G, Frank K, Groeneveld J, Jager W, Schwarz N. A framework for mapping and comparing behavioral theories in models of social-ecological systems. Ecol Econ. 2017;131:21–35. Retrieved from https://cbie.asu.edu/sites/default/files/papers/cbie_wp_2015-010.pdf.

[41] Franz MJ, Boucher JL, Evert AB. Evidence-based diabetes nutrition therapy recommendations are effective: the key is individualization. Diabetes Metab Syndr Obes. 2014;7:65–72. 10.2147/DMSO.S45140. 24591844 10.2147/DMSO.S45140 PMC3938438

[42] Powers MA, Bardsley J, Cypress M, et al. Diabetes self-management education and support in type II diabetes: A joint position statement of the American Diabetes Association, the American Association of Diabetes Educators, and the Academy of Nutrition and Dietetics. J Acad Nutr Diet. 2015;115(8):1323–34. Retrieved from https://jandonline.org/article/. 26054423 10.1016/j.jand.2015.05.012

[43] Koh HK, Blakey CR, Roper AY. Healthy People 2020: a report card on the health of the nation. JAMA. 2014;311(24):2475–6. Retrieved from https://pdfs.semanticscholar.org/45d8/0d2e4a5c90165aa97c4fe44840e51ddd5b81.pdf. 24870206 10.1001/jama.2014.6446

[44] Tucker CM, Herman KC, Pedersen TR, Higley B, Montrichard M, Ivery P. Cultural sensitivity in physician-patient relationships: perspectives of an ethnically diverse sample of low-income primary care patients. Med Care. 2003;41(7):859–70. 12835610 10.1097/00005650-200307000-00010

[45] Issaka A, Lamaro G, Renzaho A. Sociocultural factors and perceptions associated with type II diabetes among sub-Saharan African migrants in Melbourne, Victoria. Nutr Dietetics. 2016;73(1):28–35. 10.1111/1747-0080.12167.

[46] O’Reilly M, Parker N. Unsatisfactory saturation’: A critical exploration of the notion of saturation in qualitative research. Qualitative Res. 2012;13(2):190–7. 10.1177/1468794112446106.

