# Supplementary material for: Perceptions and experiences of nondiabetic Cameroonian immigrants in Minnesota on access to affordable, culturally adapted healthcare services
Source: BMC Health Serv Res. 2025 May 2;25:640. doi: 10.1186/s12913-025-12575-2 (PMC12046939; doi:10.1186/s12913-025-12575-2)
Supplement: Supplementary file 1 — Supplementary Material 1. [file 12913_2025_12575_MOESM1_ESM.docx]

Guiding Interview Questions

Research Questions RQ 1: How do nondiabetic Cameroonian immigrants perceive access to health care services for the self-management of type 2 diabetes? RQ 2: How does the consumption of traditional foods influence nondiabetic Cameroonian immigrants’ perception of type 2 diabetes? The guiding interview questions are designed to respond to the two research questions, which are further broken down into two sub-research questions. Ten interview questions are asked per sub research question to probe deeper insights into the subject matter. Subquestion 1: What is the connection made by nondiabetic individuals from Cameroon and their access to quality culturally appropriate health care services to behaviors and self-management knowledge of type 2 diabetes in Minnesota? Interview Questions 1. What does access to health care services mean to you? 2. What are your experiences with accessing health care services? 3. What does type 2 diabetes mean to you? 4. Sometimes we talk about the importance of culturally appropriate health care. What does culturally relevant mean to you? Do you think your culture is different from American culture around you? How? So, what would you recommend for culturally appropriate health care services for Cameroonians living in Minnesota? 5: What are some examples of culturally relevant health care services you have received to help you stay healthy? 5. How has your health and lifestyle improved since you started using health care services in Minnesota? If they haven’t improved, can you tell me why you think this? 6. In your opinion, what are dietary behaviors and activity behaviors? How do you think that Cameroonians dietary and activity behaviors affect the risk of type 2 diabetes? 99 7. Would you please tell a story about trying to follow health care advice on staying healthy and preventing type 2 diabetes? 8. Tell me, are you able to understand what your health care providers discuss with you about staying healthy? If yes, do you ask questions? If no, what do you do with the information when you get home? 9. Tell me, how often do you go for an office visit with your health care provider? When do you go, what are your concerns? In your opinion, are the responses understandable? If not, why not? Subquestion 2: What is the connection made by nondiabetic immigrants from Cameroon to the consumption of traditional food and knowledge of type 2 diabetes? 1. What type of food do you eat to stay healthy? 2. What type of exercise do you do to stay healthy? 3. What challenges, if any, do you face in eating healthy and staying active here in Minnesota? When planning your meals, do you ever worry about eating healthy? 4. Is it challenging to maintain a traditional diet? If yes, what might some difficulties be? If not, what makes it easy for you to follow a traditional diet? 5. What are some concerns about type 2 diabetes in your community? 6. Tell me about a time you were told to eat healthily. Who might have told you? What were some suggestions for a healthy diet? 7. Have you ever had a recommendation by a health care provider of the type of food to eat? If yes, what are some examples of food listed? Did the food listed include traditional food that you like to eat? 8. Tell me about your experience when you learned about type 2 diabetes. 9. What are you doing to prevent type 2 diabetes? 10. What effects does type 2 diabetes have on Cameroonians living in your community in Minnesota? 11. Is there anything you would like to mention that was not shared in this conversation?
